# Polymeric Nanocarriers of Drug Delivery Systems in Cancer Therapy

**DOI:** 10.3390/pharmaceutics12040298

**Published:** 2020-03-25

**Authors:** Nataša Avramović, Boris Mandić, Ana Savić-Radojević, Tatjana Simić

**Affiliations:** 1 Institute of Medical Chemistry, Faculty of Medicine, University of Belgrade, 11000 Belgrade, Serbia; 2 Faculty of Chemistry, University of Belgrade, Studentski trg 12–16, 11000 Belgrade, Serbia; borism@chem.bg.ac.rs; 3 Institute of Medical and Clinical Biochemistry, Faculty of Medicine, University of Belgrade, 11000 Belgrade, Serbia; ana.savic-radojevic@med.bg.ac.rs (A.S.-R.); tatjana.simic@med.bg.ac.rs (T.S.); 4 Serbian Academy of Sciences and Arts, 11000 Belgrade, Serbia

**Keywords:** block copolymers, polymer-drug conjugates, polymeric nanocarriers, cancer therapy

## Abstract

Conventional chemotherapy is the most common therapeutic method for treating cancer by the application of small toxic molecules thatinteract with DNA and causecell death. Unfortunately, these chemotherapeutic agents are non-selective and can damage both cancer and healthy tissues, producing diverse side effects, andthey can have a short circulation half-life and limited targeting. Many synthetic polymers have found application as nanocarriers of intelligent drug delivery systems (DDSs). Their unique physicochemical properties allow them to carry drugs with high efficiency, specificallytarget cancer tissue and control drug release. In recent years, considerable efforts have been made to design smart nanoplatforms, including amphiphilic block copolymers, polymer-drug conjugates and in particular pH- and redox-stimuli-responsive nanoparticles (NPs). This review is focused on a new generation of polymer-based DDSs with specific chemical functionalities that improve their hydrophilicity, drug loading and cellular interactions.Recentlydesigned multifunctional DDSs used in cancer therapy are highlighted in this review.

## 1. Introduction

After cardiovascular diseases, cancer is the second leading cause of death worldwide [1]. Conventional chemotherapy is the most commonly used approach in cancer treatment, along with surgery, irradiation and immunotherapy [2]. It is based on the application of small toxic chemotherapeutic molecules that interact with DNA molecules, modify them and induce cell death in cancer tissues [3,4]. Cancer cells have altered lipid and amino acid metabolic pathways, glycolysis, and redox homeostasis [1,5]. Indeed, altered energy metabolism with upregulated glucose transporter expression, disrupted redox homeostasis with upregulated glutathione transferase (GST) and high telomerase activityare responses that maintain DNA integrity, retaining replication, proliferation and cancer cell resistance [1,5,6]. Chemotherapy has many disadvantages, including drug toxicity, rapid degradation, low specificity and limited targeting. In the last few decades, nanomedicine has assumed an important role in cancer therapy based on diverse tailor-made drug delivery systems (DDSs) [7]. Nanomedicine produces materials with sizes ranging from 1–100 nm, which are used as drug nanocarriers with exceptional properties, such as their size, solubility, hydrophilicity, high specificity and a suitable drug-release profile. Nanocarriers also have an enhanced permeability and retention effect (EPR) due to their accumulation in cancer tissue with leaky vasculature [8].

Chemotherapeutics are mostly drugs that are poorly soluble in water with a limited delivery to the target tissue. Encapsulation or entrapment of drugs in nanocarriers facilitates their transport in the circulation to the cancer tissue, inhibiting their rapid biodegradation and improving their bioavailability [9]. Moreover, nanocarriers with incorporated drugs provide a longer circulation half-life of drugs, increasing their efficacy and enabling a lower dose of application [2,9]. Compared with natural polymers, synthetic nanocarriers can be tailored to control the release of encapsulated drugs by modifying their structure [10]. This review is focused on currently obtained polymer-based DDSsand it examines the challenges in improving their drug delivery properties through the introduction of targeting and stimuli-response moieties. Polymer-based drug delivery systems and polymer-drug conjugates used in cancer therapy are summarized, as well as the underlying structure responsible for the efficacy of these nanodevices.

## 2. Polymeric Nanoparticles (NPs)

Polymeric NPs are particles obtained from natural, semi-synthetic or synthetic polymers. Polymeric nanosystems are produced by a polymerization reaction of many monomer units, and under certain conditions, they can be organized and self-assemble with ananometric size (10–100 nm) [10,11,12]. Due to the high diversity of their properties, NPs attract great attention as multifunctional nanocarriers in DDSs [9,11]

**Figure 1:**
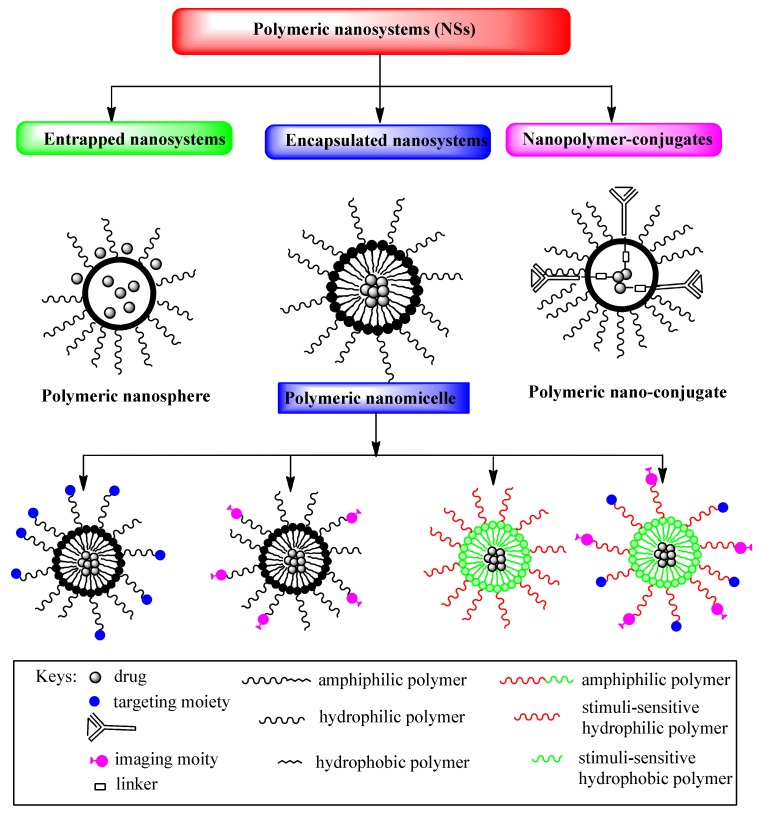
Schematic illustration of multifunctional drug delivery systems.

Depending on the preparation method, drugs can be entrapped, encapsulated or bound to polymeric NPs in the form of a nanosphere, a nanocapsule or a drug conjugate (Figure 1) [7,9,10]. Nanospheres are colloidal particles that entrap the drug inside their matrix by physical dispersion or by adsorption on the particle surface, while nanocapsules are systems consisting of a core cavity with an encapsulated drug and polymeric shell surrounding it. Polymeric capsules can be designed by the conjugation of targeting ligands that increase selectivity for cancer cells and improve intracellular drug delivery, as well as reducing different side effects and drug toxicity. Targeting ligands of polymeric capsules are commonly monoclonal antibodies (mAbs) or antibody fragments, aptamers, peptides and small molecules, such as folic acid, which are conjugated to the shell-forming block [13,14,15,16,17,18,19]. These ligands are specifically bound to antigens or receptors that are overexpressed on the cancer cell [20] and they enable cellular selectivity and intracellular delivery of polymeric micelles [13]. Different designed polymeric capsules suitable for targeting the release of drugs are shown in Figure 1. The efficacy of polymeric carriers modified with targeting ligands depends on the ligand properties, such as their density and binding affinities to receptors, which can enhance receptor internalization and the biodistribution of drugs. Drug-conjugates have a drug that is chemically bonded to the polymer through a linker/spacer. The bond drug-linker/spacer is a common breakage-point when the drug is released at the target site (Figure 1).

**Figure 2:**
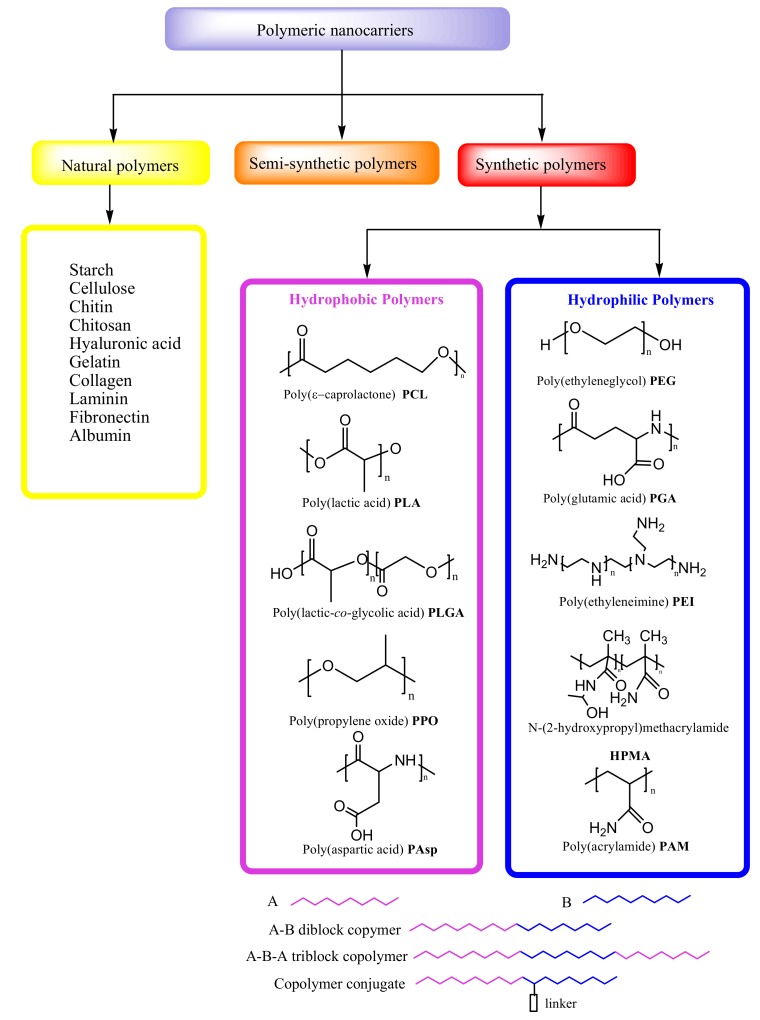
Types of polymeric nanocarriers.

Natural polymers are biopolymers, including different classes of polysaccharides and proteins, which, due to their biocompatibility and biodegradability, are particularly suitable for medical applications, as in cell-based transplantation, tissue engineering and gene therapy [10] (Figure 2). Natural polymers can be combined with synthetic molecules through the chemical modification of their functional groups and so-called semi-synthetic polymers can mimic human tissue components. In formulations of controlled DDSs, synthetic polymers attract more attention than biopolymers due to the considerable potential for the design of their structure and modifications of their physicochemical properties (Figure 2) [8]. Synthetic polymeric micelles exhibita high capacityto incorporate a broad range of bioactive molecules, such as antisense oligonucleotides [21], plasmid DNA [22], proteins [23], small interfering ribonucleic acids (siRNAs) [24], messenger RNAs (mRNAs) [25] and photosensitizers [26], by tailoring the core-forming segments of the block copolymers. In fact, several poly-ion complex (PIC) micelles have been designed that incorporate negatively charged biomolecules by electrostatic interaction with positively charged block copolymers [21,27]. In addition, they can be stabilized by the covalent crosslinking of their core through disulfide bonds [28], which can be cleaved under specific intracellular conditions, enabling the complexes to escape from endosomal compartments after endocytosis and to deliver the biomolecules to subcellular destinations [29] without drug degradation. By introducing hydrophobic molecules such as cholesterol to the core [30], PIC micelles become more stable, with a longer half-life in the bloodstream, allowing for the delivery of intact biomolecules to therapeutic targets. PIC micelles obtained from block copolymers with a core-forming polycation such as polyaspartamides, support enhanced delivery of biomacromolecules to the cytosol of cells, and the gene transfection in vitro and in vivo [25,29,30,31,32,33,34,35].

In recent years, the great potential of synthetic polymers as drug carriers has been highlighted, particularly because of the possibility to develop DDSs with a target sustained/controlled release of drugs [1]. The encapsulation of cancer drugs in polymeric micelles with modifications for cancer targeting and triggered release results inmore efficient drug delivery (Figure 1).

In addition to biocompatibility and biodegradability, synthetic polymers used in DDSs should be activated at the site of action, to be stable in blood circulation, to have low toxicity and immunogenicity, and to provide protection fromthe degradation of drugs before the target tissue is reached. Additionally, it is necessary that polymer nanocarriers of DDSscan be easily synthesized without impurities [8].

## 3. Amphiphilic Block Copolymers as Carriers in Drug Delivery Systems

### 3.1. Hydrophobic and Hydrophilic Polymeric Nanocarriers

Polymeric micelles are the most common nanocarriers of DDSs asregards the original core-shell structure [8]. They consist of amphiphilic block copolymers with hydrophilic and hydrophobic units that self-assemble in water solution at the critical micelle concentration (CMC). Micellar polymeric units can be formed in different ways, such as diblock copolymers (A-B), triblock copolymers (A-B-A) and copolymer conjugates (Figure 2) [9].

The hydrophobic core is suitable for encapsulating poorly water-soluble drugs, and the pharmacokinetics of drug release can be controlled by its modification. The most frequently used hydrophobic polymers for core formation of NPs are: poly(ε-caprolactone) (PCL), poly(lactic acid) (PLA), poly(lactic-*co*-glycolic acid) (PLGA), poly(propylene oxide) (PPO) and poly(aspartic acid) (PAsp) (Figure 2). The hydrophilic polymers that are most frequently considered for the hydrophilic shell of NPs in DDS include poly(ethylene glycol) (PEG), poly(glutamic acid) (PGA), poly(ethyleneimine) (PEI), N-(2-hydroxypropyl)methacrylamide (HPMA) and poly(acrylamide) (PAM) (Figure 2). A frequently used hydrophilic polymer of DDSs is PEG, which providesdistinctstability to NPs due to the reduction of nonspecific interactions with blood proteins, thus preventing their aggregation [36].

### 3.2. Block Copolymers of DDSs in Cancer Therapy

Poly(ethylene glycol)-b-poly(ε-caprolactone)(PEG-PCL) is a polyether-polyester diblock copolymer, synthesized by ring-opening polymerization of ε-caprolactone and PEG [37]. It is suitable for a variety of DDSs because ofits high biocompatibility, biodegradability and low toxicity. Many DDSs based on PEG-PCL with different hydrophilic/hydrophobic ratios (PEG/PCL) have been obtained, enablinghigher cellular internalization byincreasingPEGcontribution(PEG/PCL = 5/5) [38]. Çırpanlı et al. have recently developed PEG-PCL nanocarriers for the controlled delivery of camptothecin (CPT), whose active lactone form was maintained by drug entrapment to hydrophobic PCL, preventing drug hydrolysis in the carboxylate inactive form (Table 1) [39]. Furthermore, Hu et al.have designed a nanoplatform with paclitaxel (PTX) encapsulated in a triblock PCL-PEG-PCL copolymer that in combination with circadian chrono-modulated chemotherapy confirmed sustained drug release and a lower cytotoxic effect compared with free PTX injection [40]. Hong et al. obtained image-guided polymeric micelles, including a folate-conjugated PEG-b-PCL copolymer loaded with doxorubicin (DOX) and superparamagnetic iron oxide nanoparticles (SPIONs) [41]. Active targeting was achieved by the conjugation of folic acid to the PEG-b-PCL shell-forming block, allowing micelles to specifically bind to receptors for folic acid that are overexpressed on the tumor cells. Drug-delivery efficiency and diagnostics were considerably improved by the combination of active tumor targeting and imaging in human hepatic carcinoma cells (Bel 7402 cells). Bel 7402 cells overexpress surface receptors for folic acid that bind these folate-conjugated polymeric micelles, providing targeted delivery of DOX to the cancer cells and exhibiting high inhibition of proliferation as compared to non-targeted micelles. The epidermal growth factor receptor (EGFR) is a transmembrane glycoprotein with an intracellular tyrosine kinase domain, which is overexpressed on the cells of solid cancers [42]. Lee et al. developed EGF-receptor-targeted PEG-b-PCL micelles with incorporated DOX and labeled with ^111^In. Images were taken with micro-SPECT/CT intratumoral distribution of both targeted and non-targeted micelles confirmedenhanced accumulation in tumor tissue with the targeted micelles (T-BCM) as compared to non-targeted micelles (NT-BCM) [43].

Guo et al. have demonstratedthe suitability of the hydrophobic polymer PLGA to encapsulate the low-solubility drug PTX in a poly(lactic-co-glycolic acid)-poly(ethylene glycol) (PLGA-PEG) nanoplatform [44]. A longer circulation time and increased cancer inhibition were confirmed when this DDS was decorated with DNA aptamersbecause of the enhanced cellular association of NPs in C6 glioma cells (Table 1) [44]. Recently, the Shafiei–Irannejadgroupdeveloped a poly(lactic-co-glycolic acid)-d-α-tocopheryl polyethylene glycol 1000 succinate (PLGA-TPGS) nanodevice with two encapsulated drugs, doxorubicin (DOX) and metformin (Met), which was shown to successfully inhibit the P-glycoprotein efflux system through the combined effects of Met and TPGS.This DDSis mostly used in the treatmentof multidrug-resistant breast cancer (Table 1) [45]. Examination of the outcome of delivery of the combination of DOX and PCT applied in a polymeric system, PEG-PLGA, has attracted attention due to the observed synergistic anticancer effect of drugs that achieved an improved/higher therapeutic outcome. Wang et al. reported that methoxy PEG-PLGA NP co-loaded with hydrophilic DOX and hydrophobic PCT possessed greater cancer growth inhibition than polymeric micelles loaded with only one drug (either DOX or PCT), with the highest anticancer efficacy at a concentration ratio of 2:1 [46].Xu et al. also obtained an amphiphilic poly(ethylene glycol)-poly(L-lactic acid) (PEG-PLA) diblock copolymer with incorporated DOX and PCT at a molar ratio of 1:1 in ultrafine PEG-PLA fibers, using an “emulsion-electron spinning” method [47].These polymeric micelles revealed lower cell viability and a higher percentage of cell-cycle arrest inrat glioma C6 cells 72 h after treatment. Moreover, Duong et al. also prepared a PEG-PLGA copolymer system for delivery of both DOX and PCT, including targeting ligand folate (FOL) and TAT peptide, which enhances the cellular interaction between PEG-PLGA micelles ina human oral cavity carcinoma KB cell line [48]. Essentially, FOL increases the targeting ability of the drug carriers, while TAT peptide is a cell-penetrating peptide (CPP) employed formodification of the carrier surface. Different concentration ratios of DOX and PCT were applied in PEG-PLGA micelles, and higher effectiveness was reached at a concentration ratio of 1:0.2 than at a concentration ratio of 1:1 [48]. The same ratio of drugs (1:0.2) was used in the study by Lv et al. on a deoxycholate decorated methoxy poly(ethylene glycol)-b-poly(l-glutamic acid)-b-poly(l-lysine) triblock system (mPEG-b-PLG-b-PLL) used for delivery of DOX and PCT, and the synergistic effect of the drugs was confirmed [49]. Namely, the amphiphilic triblock copolymer spontaneously self-assembled in a water solution into polymeric micelles, forming three different domains with diverse functions: the outer hydrophilic PEG corona enabled extended circulation, the middle hydrophilic PLG shell loaded DOX by electrostatic interactions, and the inner hydrophobic PLL core incorporated PCT by hydrophobic interactions. Efficient tumor growth inhibition was obtained for this co-delivery system in an A549 xenograft tumor model that caused 3.2-, 6.3-and 2.4-fold decreases in tumor volume than when treated with free DOX, free PCT and free DOX + PCT, respectively. Theresults obtained by studying the synergistic effect of DOX and PCT with various drug combinations suggest that control of the applied amount of DOX is linked to its more rapid release than that of PCT; the release of DOX facilitates the release of PCT, providing synergistic action.

A phase I study of the poly(ethylene glycol)-b-poly(glutamic acid) (PEG-PGlu) nanocarrier, which consisted of a hydrophilic shell of PEG, a hydrophobic core of the derivative of PGlu and incorporated drug cisplatin, has confirmed lower toxicity and completely different pharmacokinetics than those for free cisplatin [50,51,52]. The clinical study of PEG-PGlu(Cisplatin) NPs in combination with gemcitabine has entered phase III in patients with metastatic pancreatic cancer (Table 1) [52]. EGFR, known as a transmembrane glycoprotein, that is overexpressed on the cells of solid cancers [42], is targeted with the monoclonal antibody mAbC225. Vega et al. prepared mAb C225-based targeting PEG-b-PGlu micelles with loaded DOXthat displayed a higher anticancer effect than free DOX, observed as inhibition of the growth of A431 cells [53]. 

DOX is a pro-apoptotic drug causing DNA damage and the activation of apoptosis [76] while curcumin (CUR) is an antiangiogenic agent that blocks the transcription factor NF-jB, thus targeting the MAPK and PI3K/PKB pathways and inhibiting protein kinase-C [77,78]. The co-delivery of multiple drugs with the complementary anticancer mechanisms of nanocarriers offers an effective strategy to treat cancer. To date, several NP formulations, such asmPEG-PCL micelles [79], poly(alkyl cyanoacrylate) NPs [80] and PLLA NPs [81], have been obtained for the co-delivery of DOX and CUR, although the majority of these NPs release the drug non-specifically, with a slow degradation of the polymeric micelles. Co-encapsulation of DOX and CUR in pH-sensitive NPs has been shown as a good strategy in treating cancer in a synergistic manner with increased efficacy and lower toxicity by off-target exposure.The new synthesized triblock copolymer monomethoxy (polyethylene glycol)-b-poly(D,L-lactic-co-glycolic acid)-b-poly(L-glutamic acid) (mPEG-PLGA-PGlu), including two hydrophobic polymers, PGlu and PLGA, and the modified hydrophilic mPEG polymer, confirmed possible encapsulation of CUR and DOX, with simultaneous targeting of heterogeneous breast cancer cells (Table 1) [54]. These NPs are pH sensitive, and the PGlu segment incorporates DOX through electrostatic interactions that alter the configuration in response to endosomal pH, while PLGA encapsulates CUR inside the core through hydrophobic interactions. The loading efficiencies were 80.30% and 96.2% for CUR and DOX, respectively. A cascade of sustainedrelease, first of CUR followed by a slower release of DOX, was shown at physiological pH.

The micellar PTX formulation PEG-PAsp, also known as NK105, consists of PEG and modified polyaspartate as the hydrophobic block, with PTX incorporated via hydrophobic interactions. In order to increase hydrophobicity and improve drug incorporation, half of the carboxylic groups of the polyaspartate block were esterified with 4-phenyl-1-butanol after treatment with the condensing agent 1,3-diisopropylcarbodiimide [55,56,57]. The paclitaxel (PCX) formulation of the poly(ethylene glycol)-b-poly(aspartic acid) (PEG-PAsp) nanoplatform exhibited similar cytotoxicity in lung, gastric, colon and ovarian cancers as the free drug, as well as higher resistance to allergic reactions in a phase I clinical study [55,56,57].A phase II study is ongoing with patients with advanced stomach cancer (Table 1). Vilar et al. reported a similar DOX formulation of an amphiphilic poly(ethylene oxide)-b-poly(aspartic acid)(PEO-b-PAsp) copolymer, in which the hydrophilic polymer PEG was substituted with PEO [57]. In phase I clinical trials, the PEO-b-PAsp(DOX) formulation displayed higher anticancer activity than free DOX, with a maximum tolerated dose of 67 mg/m^2^.

Pluronic is a triblock non-ionic PEO-PPO-PEO copolymer composed of poly(ethylene oxide) (PEO) and poly(propylene oxide) (PPO) copolymers. A DOX formulation of the Pluronic PEO-PPO-PEO(DOX) device, used as a P-glycoprotein inhibitor, showed a higher anticancer effect than free DOX in phase II clinical trials [59]. In the Pluronic PEO-PPO-PEO(DOX) formulation, also known as SP1049C, DOX is encapsulated by noncovalent bonds in the hydrophobic core of the micelles [82]. A study is ongoing with patients with metastatic esophageal adenocarcinoma, gastric and stomach cancers in phase III clinical trials [60]. Wang et al. obtained folate-targeted micelles composed of Pluronic copolymers, P105 and P105/L101, with loaded PTX in the core; they exhibited increased internalization that explained the improved cytotoxicity of PTX to tumor cells in MCF-7 and MCF-7/ADR cells [83]. Chen et al. produced atriblock co-delivery system for both DOX and PCT, which consists of conjugated Pluronic P105 with DOX and encapsulated PCT into a hydrophobic core formed by P105-DOX and Pluronic F127. The in vivo study of these Pluronic micelles applied in MCF7/ADR and KBv cell lines described eficient tumor growth inhibition over 14 days at a 2:3 ratio of the drugs [84]. Moreover, Ma et al. studied the application of pH-sensitive Pluronic F127-grafted chitosan for delivery of DOX together with PCT in vivo, with a concentration ratio of 1:1 for DOX and PCT [85]. 

Recently, delivery of both DOX and PTX was studied using double-reacting nanoparticles built of four polymers, such as poly(DL-lactide-co-glycolide) (PLGA), Pluronic F127 (PF127), chitosan, and hyaluronic acid (HA). PLGA in combination with PF127 formed more stable and homogeneous nanoparticles than PLGA or PF127 alone. HA was used as a targeting ligand in cancer stem cells to reduce drug resistance. The anticancer effect of these micelles co-loaded with both drugs was amplified ~500 times as compared to a simple mix of the two drugs [86]. 

Hu et al. have reported a multifunctional DDS with oxygen-generating theranostic nanoparticles (CDM NPs) composed of the light-activated photosensitizer chlorin e6 (Ce6),DOX and colloidal manganese dioxide (MnO_2_), assembled with the triblock poly(ε-caprolactone-co-lactide)-b-poly(ethylene glycol)-b-poly(ε-caprolactone-co-lactide) PCLA-PEG-PCLA copolymer and used in breast cancer therapy [61]. Combined chemotherapy with PDT could overcome the disadvantages of both therapies. In addition to the well-known side effects of chemotherapy, the efficacy of PDT is dependent on hypoxia in cancer cells. MnO_2_ in NPs reduces cancer hypoxia by catalyzing the decomposition of endogenous hydrogen peroxide (H_2_O_2_), producing O_2_, andfurther, by the light-activation of chlorin e6 (Ce6), it produces cytotoxic singlet oxygen (^1^O_2_), responsible for the killing of cancer cells. These theranostic CDM NPs have high stability and biocompatibility, both in vitro and in vivo, indicating that combined chemotherapy-PDT could attract great attention in future studies.

Nucleic acid therapeutics, such as siRNA, inhibit cancer-associated proteins or activate cancer-suppressing pathways [76]. However, siRNA is very unstable in the systemic circulation and poorly penetrates the cell membrane due to its high molecular weight, large negative charge and enzyme-induced degradation. Therefore, combinations of chemotherapeutics with siRNA, complexed by cationic polymeric copolymers, are under examination [24]. Cationic charged polymers, such as poly(ethyleneimine) (PEI) and poly[2-(N,N-dimethylaminoethyl) methacrylate] (PDMAEMA) [29,30] are suitable for complex binding siRNA by electrostatic interaction, preventing the degradation of siRNA and enhancing its delivery across the cell membrane. Jin et al. have recently developed a promising smart delivery system composed ofthe cationic deblock poly(ethyleneimine)-poly(lactic acid) (PEI-PLA) copolymer, designed to deliver the drug PTX and the siRNA with a synergistic strategy in chemo- or gene therapy in the treatment of non-small cell lung cancer (Table 1) [61]. This PTX formulation of NPs increases the effect of the drugby siRNA inhibition of target proteins responsible for modulating cancer cell metabolism and proliferation. This co-delivery system is a promising DDS with regard to high drug loading, a longer half-life in the circulation, lower toxicity and an antiproliferative effect of PTX on A549 cells. Cao et al. conjugated PEI with poly(ε-caprolactone) (PCL) through disulfide or ester covalent linkages and DOX was loaded into PEI-PCL micelles by complex binding of the pro-apoptotic protein siBcl-2 [33]. This DOX and Bcl-2 siRNA co-delivery system induced a 60% decrease in cell viability 96 h after treatment in Bel7402 cell lines. When siScramble was used instead of siBcl-2,the synergistic effect of the co-delivery of DOX and siBcl-2 was confirmed sincecell viability decreased by only 40%. With further modification of the DOX+siBcl-2 nanocarrier by conjugation with folic acid, cell viability decreased to 5%. Navarro et al. obtained the amphiphilic copolymer PEI conjugated with a dioleoylphosphatidylethanolamine (DOPE) moiety with the delivery of P-glycoprotein siRNA (siP-gp) and DOX that reversed the multidrug resistance in MCF7/ADR cells [34]. Another co-delivery system for siRNA and chemotherapeutic DOX based on PEI with attached stearic acid as a hydrophobic compartment (PEI-SA) was reported by Huang et al. [35]. The combination of DOX and VEGF siRNA (siVEGF) co-delivered by PEI-SA micelles demonstrateda high in vivocancer growth inhibition effect in Huh-7 cells. The amphiphilic structure of PEI-SA micelles supportsmultifunctional tasks for both genetic and chemotherapeutic application. The hydrophilic shell modified with cationic charges of PEI-SA and DNA or with anionic charges of siRNA are bound by electrostatic interaction, while the hydrophobic core encapsulates the hydrophobic drug. Tang et al. also produced an amphiphilic copolymer based on PEI and poly((1,4-butanediol)-diacrylate-b-5-hydroxyamylamine) (PDHA) [87]. When this copolymer, polyethyleneimine-block-poly((1,4-butanediol)-diacrylate-b-5-hydroxyamylamine) (PEI-PDHA), was self-assembled with PEG, it formed micelles that can co-load with three agents, siSnail, siTwist and the drug PCT, exhibiting a 54.7-fold lower value of IC50 than that of free PCT.

### 3.3. Polymer-Drug Conjugates in Cancer Therapy

Some PEG- and HPMA-drug conjugates are approved by the FDA and have found clinical application due to their excellent properties, such as good solubility in water and many organic solvents, high hydrophilicity, low toxicity and immunogenicity [88,89]. Several important polymer-drug conjugates used in cancer therapy are summarized in Table 1.

PEG-based conjugates with the drugs camptothecin (CPT), camptothecin derivatives, SN38 and irinotecan (C-11), used as topoisomerase I inhibitors, have progressed to phases I and II of clinical studies [63,64,65]. These PEG-based conjugates carry the drugs in a lactone-active form bonded to PEG through a glycine spacer. The PEG-CPT conjugate known as prothecan displayed low toxicity and satisfactory tolerance in phase II studies in patients with adenocarcinoma of the stomach and the gastroesophageal (GE)junction, although it confirmed significantly lower drug loading (1.7%) than other polymer-drug conjugates [63]. PEG-SN38 (EZN-2208) and PEG-irinotecan (NKTR-102) conjugates were synthesized by coupling a 4 arm PEG of 40 kDa with SN38 and irinotecan, respectively [66,67]. Irinotecan is a derivative of SN38 containing an additional bis-piperidine group, which the PEG-irinotecan conjugate releases at the targeting site of action, forming the active metabolite SN-38. However, the PEG-SN38 conjugate provided a longer half-life of SN38 in the circulation and it is up to a 245-fold more efficient thanPEG-irinotecan conjugate in human cancer cell lines [67]. The anticancer activity of PEG-SN38 was revealed in the therapy of breast, colorectal and pancreatic cancers. The PEG-irinotecanconjugate had a half-life of 15 days compared to 4 h for free irinotecan [75]. The PEG-irinotecan conjugate combined with cetuximab has entered into phase II study in the therapy of ovarian, breast, colorectal and cervical cancers [68].

Copolymers of HPMA developed by Kopecek et al. [45] showed excellent hydrophilic properties as biocompatible carriers for the delivery of drugs such DOX [69,70] and diaminocyclohexane (DACH)-platinum (Table 1) [72,73,74,75]. Indeed, the developed HPMA-DOX and HPMA-DACH platinum conjugates have drugs bonded to copolymers via an amidebond withthe tetrapeptide glycine-phenylalanine-leucine-glycine (GFLG) and a pH-sensitive linker, respectively. The HPMA-DOX conjugate (PK1, FCE28068), with DOX bound to the copolymer across the C-terminus of the glycine moiety of linker GFLG, is decomposed by thiol-dependent lysosomal proteases [69,70]. It is the first water-soluble drug conjugate under clinical assessment; it exhibited a 5-fold higher maximum tolerated dose (MTD) of 320 mg/m^2^ than the free drug in a phase I study [69]. Clinical studies revealed its anticancer efficacy in therapy of breast, colorectal and non-small cell lung cancers [70]. On the other hand, the HPMA-(DACH)-platinum conjugate known as AP-5346, was developed [72,73,74,75]. It allows controlled release of the drug in cancer cells by passive transport due to the small size of NPs (25 kDa) with a longer half-life than the free (DACH)-platinum drug. Its anticancer activity was studied in metastatic melanoma and ovarian cancer in a phase I study, and currently, with the name Prolindac, it is undergoing phase II clinical trials [75]. Furthermore, based on molecular weight, chemical structure and loading efficacy similar to the HPMA-DOX conjugate, the HPMA-DOX-galactosamine system was developed; it possesses a conjugated galactosamine specified to target liver cancer cells [90,91,92]. This conjugate showed a 2-fold lower MTD (160 mg/m^2^) than HPMA-DOX, although in II phase trials adose of 120 mg/m^2^ was evaluated because of its toxicity, which produces fatigue and leads to neutropenia and mucositis in patients with metastatic liver cancer [92].Moreover, in phase I clinical trials, HPMA-camptothecin (CPT) and -paclitaxel (PCX) conjugates with drugs bonded via an ester bond through a linker, were also studied; they showed high toxicity due to the rapid ester hydrolysis and drug release in vivo [71].

## 4. Stimuli-Responsive Polymer-Drug Conjugates

In the last two decades, many smart nanoplatforms have been designed by introducing stimuli and targeting moieties into polymers of DDSs [93,94,95,96,97]. Some pH- and redox-responsive polymeric NPs are described here as the most commonly used triggers in DDSs.

**Figure 3:**
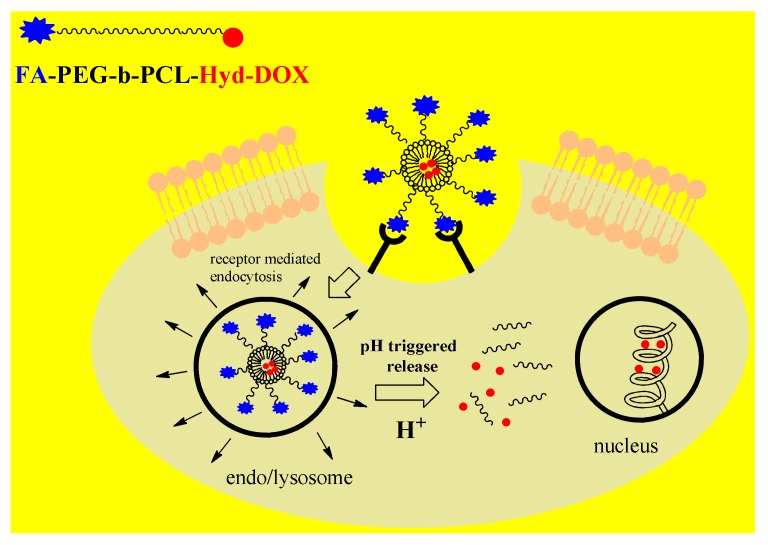
Mechanism of action of pH-responsive polymer nanoparticles(NPs) decorated with targeting ligand folic acid (FA) and with the drug doxorubicin(DOX)bound via a hydrazone bond to diblock copolymerPoly(ethylene glycol)-b-poly(ε-caprolactone)(PEG-PCL).

Due to increased aerobic glycolysis, cancer cells have an acidic environment (pH 6.5–7.2), in particular the intracellular organelles such as endosomes (pH 5–6) and lysosomes (pH 4–5) [93]. Therefore, many pH-responsive DDSs were designed by covalent attachment of the drug to the polymeric NPs via an acid-labile bond. Bae et al. created diblock PEG-b-Pasp copolymers conjugated with the drug adriamycin via a hydrazone bond, which can be easily cleaved under the acidic conditions found in cancer cells, followed by rapid release of the drug and break-up of copolymers [93]. The hydrazone bond of diverse nanoplatforms has been successfully applied to transport different anticancer drugs such as DOX, PCX and cisplatin [8]. Guo et al. designed a multifunctional polymeric-drug conjugate, FA-PEG-b-PCL-hyd-DOX, with a diblock PEG-PCL copolymer bound to DOX via a labile hydrazone bond, and decorated with folic acid (FA) [8]. This DDS exerts cancer targeting ability through the folate receptor (FR) interfering with endocytosis and pH-triggered drug release by the breaking of the hydrazine bond under acidic conditions (Figure 3).

Xiong et al.recently reported on a multifunctional pH-responsive DDS composed of the diblock PEO-b-PCL copolymer and the αvβ3 integrin-targeting ligand, RGD4C, on the micellar surface [94,95]. The delivery systems are based on RGD4C-PEO-b-P (CL-Hyd-DOX) micelles, including the hydrophobic core (PCL), the drug DOX incorporated in the core via a pH-labile hydrazonebond, and a hydrophilic shell (PEO) decorated with the targeting peptide ligand RGD4C. This multifunctional DDS had the highest cytotoxic response in DOX-sensitive cancer cells, while the mitochondria-targeted NPs, created as RGD4C-PEO-b-P (CL-Ami-DOX) micelles, conjugated with DOX via a stable amide bond (Ami), showed the highest cytotoxic response in DOX-resistant cancer cells.

**Figure 4:**
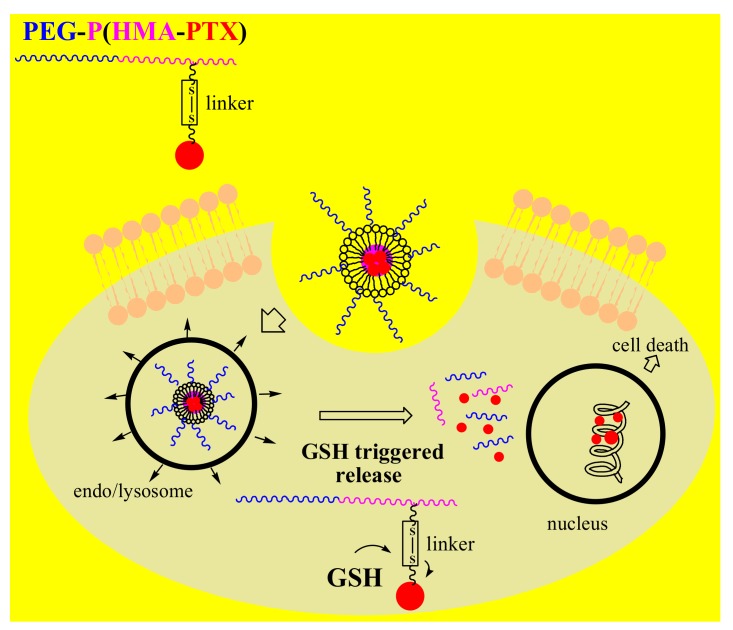
Mechanism of action of redox-responsive polymernanoparticles (NPs) with bonded drug PTX via a disulfide linker to diblock copolymerPoly(ethylene glycol)-b-poly(2-hydroxyethyl methacrylate)(PEG-b-PHEMA).

Due to the increased concentration of glutathione (GSH)in the cytosol and subcellular organelles, there is a redox potential difference between the intra- and extracellular micro environments of normal cellsthat is larger in cancer cells because of the 2- to 4-fold higher concentration of GSH [8]. The significant increase in the intracellular redox potential was used as an internal stimulus for creating intelligent DDSs by introducing a redox-sensitive functional group into the polymeric nanocarriers or reducible linker. Similar to the previously described polymeric-drug conjugate, FA-PEG-b-PCL-hyd-DOX, Shi et al. designed a redox-responsive FA-PECL_SS_-DOX nanocomplex by linking PEG and PCL polymers via a redox-sensitive disulfide bond [96]. Thus, as a result of stimulation by the acidic and reducing medium of cancer cells, GSH triggered DOX release by breaking the disulfide bond, with the drug efficiently killing the cancer cells. Recent studies have also introduced a novel multifunctional redox-responsive DDS based on the amphiphilic diblock PEG-b-PHEMA copolymer and covalently bounded PTX via a disulfide linker to poly(2-hydroxyethyl methacrylate)PHEMA in NPs (Figure 4) [97]. This biocompatible polymer-drug conjugate, PEG-b-P(HEMA-PTX),possessedglutathione-dependent cytotoxicity, providing higher proliferation inhibition in glutathione monoester-pretreated HeLa cells than in non-pretreated HeLa cells. 

## 5. Conclusions 

This article reviews recently obtained polymeric DDSs with applications in cancer therapy. PEG and HMPA block copolymers, polymer-based drug conjugates and some multifunctional DDSs decorated with stimuli and targeting moieties are summarized and their higher anticancer efficacy compared to administered free drugs is highlighted. Diverse polymeric co-delivery systems have been obtained showing high anticancer efficacy, especially in multidrug-resistant cancers. Polymeric block micelles are ideal platforms for the co-delivery of combined drugs due to their multicompartment structure. A single polymeric delivery system with two co-loaded drugs has a promising role in cancer therapy because ofthe simultaneous targeting of different multidrug-resistance mechanisms. Moreover, the ratio between two applied drugs can directly influence the efficacy of a co-delivery system. Additionally, chemotherapy by the application of different polymeric micelles combined with gene therapy has attracted considerable attention. Ligand-modified polymeric micelles improve the anticancer effect of drugs because of receptor-mediated transport mechanisms. Efficient therapeutic DDSs have been obtained by developing sophisticated polymeric micelles that can encapsulate various bioactive molecules. Polymeric nanoplatforms have a promising role in cancer chemotherapy, although moreclinical studies are necessaryin order to better understand their benefits and drawbacks.

## Figures and Tables

**Table 1:** Polymeric-anticancer drug nanoparticles (NPs), their loading mode and function.

Polymer	Drug	Loading Mode	Function	Reference
PEG-PCL	Camptothecin (CPT)	Entrapment	Colon, breast, ovarian, lung and brain cancers	[39]
PCL-PEG-PCL	Paclitaxel (PTX)	Encapsulation	Lung cancers in combination with chrono-modulated chemotherapy	[40]
PLGA-PEG	Paclitaxel (PTX)	Encapsulation	Breast, pancreatic and ovarian and brain cancers	[44]
PLGA-TPGS	Doxorubicin(DOX)- Metformin (Met)	Encapsulation	Multidrug resistance P388 cancer cell lines	[45]
PEG-PGlu	Cisplatin	Encapsulation	Solid cancers	[50,51,52]
mPEG-PLGA-PGlu	Doxorubicin(DOX)	Encapsulation	Breast cancer	[54]
PEG-PAsp	Paclitaxel (PTX)	Entrapment	Advanced stomach cancer	[55,56,57]
PEO-b-PAsp	Doxorubicin	Entrapment	Pancreatic cancer	[58]
PEO-PPO-PEO	Doxorubicin.	Encapsulation	Metastatic adenocarcinoma of the esophagus and gastroesophageal junction	[59,60]
PCLLA-PEG-PCLLA	Doxorubicin (DOX)	Encapsulation	Breast cancer	[61]
PEI-PLA	Paclitaxel (PTX)	Entrapment	Lung cancer	[62]
PEG	Camptothecin (CPT)SN38 Irinotecan (C-11)	Copolymer-drug conjugation	Colorectal, metastatic breast cancer, platinum-resistant ovarian cancer and metastatic cervical cancer	[63,64,65,66,67,68]
HPMA	Doxorubicin (DOX)	Copolymer-drug conjugation	Lung and breast cancer	[69,70]
HPMA	Paclitaxel (PTX)	Copolymer-drug conjugation	Solid cancers	[71]
HPMA	Diaminocyclohexane(DACH)-platinum	Copolymer-drug conjugation	Solid cancer, ovarian cancer	[72,73,74,75]

## References

[1] Tran S., DeGiovanni P.J., Piel B., Rai P. (2017). Cancer nanomedicine: A review of recent success in drug delivery. Clin. Transl. Med..

[2] Bahrami B., Hojjat-Farsangi M., Mohammadi H., Anvari E., Ghalamfarsa G., Yousefi M., Jadidi-Niaragh F. (2017). Nanoparticles and targeted drug delivery in cancer therapy. Immunol. Lett..

[3] Pérez-Herrero E., Fernández-Medarde A. (2015). Advanced targeted therapies in cancer: Drug nanocarriers, the future of chemotherapy. Eur. J. Pharm. Biopharm..

[4] Avramović N., Ignjatović N., Savić A. (2019). Platinum and ruthenium complexes as promising molecules in cancer therapy. Srp. Arh. Celok. Lek..

[5] Escobar Q.M., Maschietto M., Krepischi A.C.V., Avramovic N., Tasic L. (2019). Insights into the Chemical Biology of Childhood Embryonal Solid Tumors by NMR-Based Metabolomics. Biomolecules.

[6] Radic T., Coric V., Bukumiric Z., Pljesa-Ercegovac M., Djukic T., Avramovic N., Matic M., Mihailovic S., Dragicevic D., Dzamic Z. (2019). GSTO1*CC Genotype (rs4925) Predicts Shorter Survival in Clear Cell Renal Cell Carcinoma Male Patients. Cancers.

[7] Hossen S., Hossain K.M., Basher M.K., Mia M.N.H., Rahman M.T., Uddin J.M. (2019). Smart nanocarrier-based drug delivery systems for cancer therapy andtoxicity studies: A review. J. Adv. Res..

[8] Guo X., Wang L., Wei X., Zhou S. (2016). Polymer-Based Drug Delivery Systems for Cancer Treatment. J. Polym. Sci. A Polym. Chem..

[9] Parveen S., Arjmand F., Tabassum S. (2019). Clinical developments of antitumor polymertherapeutics. RSCAdv..

[10] Fathi M., Barar J. (2017). Perspective highlights on biodegradable polymeric nanosystems for targeted therapy of solid tumors. Bioimpacts.

[11] Calzoni E., Cesaretti A., Polchi A., Di Michele A., Tancini B., Emiliani C. (2019). Biocompatible Polymer Nanoparticles for Drug Delivery Applications in Cancer and Neurodegenerative Disorder Therapies. J. Funct. Biomater..

[12] Cabral H., Kataoka K. (2014). Progress of drug-loaded polymeric micelles into clinical studies. J. Control. Release.

[13] Allen T.M. (2002). Ligand-targeted therapeutics in anticancer therapy. Nat. Rev. Cancer.

[14] Torchilin V. (2008). Antibody-modified liposomes for cancer chemotherapy. Expert Opin. Drug Deliv..

[15] Cheng W.W., Allen T.M. (2010). The use of single chain Fv as targeting agents for immunoliposomes: An update on immunoliposomal drugs for cancer treatment. Expert Opin. Drug Deliv..

[16] Farokhzad O.C., Cheng J., Teply B.A., Sherifi I., Jon S., Kantoff P.W., Richie J.P., Langer R. (2006). Targeted nanoparticle-aptamer bioconjugates for cancer chemotherapyin vivo. Proc. Natl. Acad. Sci. USA.

[17] Oba M., Vachutinsky Y., Miyata K., Kano M.R., Ikeda S., Nishiyama N., Itaka K., Miyazono K., Koyama H., Kataoka K. (2010). Antiangiogenic gene therapy of solid tumor bysystemic injection of polyplex micelles loading plasmid DNA encoding soluble flt-1. Mol. Pharm..

[18] Miura Y., Takenaka T., Toh K., Wu S., Nishihara H., Kano M.R., Ino Y., Nomoto T., Matsumoto Y., Koyama H. (2013). Cyclic RGD-linked polymeric micelles for targeted delivery of platinum anticancer drugsto glioblastoma through the blood-brain tumor barrier. ACS Nano.

[19] Bae Y., Jang W.D., Nishiyama N., Fukushima S., Kataoka K. (2005). Multifunctional poly-meric micelles with folate-mediated cancer cell targeting and pH-triggered drug releasing properties for active intracellular drug delivery. Mol. Biosyst..

[20] Torchilin V.P. (2008). Cell penetrating peptide-modified pharmaceuticalnanocarriers for intracellular drug and gene delivery. Biopolymers.

[21] Skatrud P.L. (2002). The impact of multiple drug resistance (MDR) proteins on chemotherapy and drug discovery. Prog. Drug Res..

[22] Dai X., Tan C. (2015). Combination of microRNA therapeutics with small-molecule anticancer drugs: Mechanismof action and co-delivery nanocarriers. Adv. Drug Deliv. Rev..

[23] Teo P.Y., Cheng W., Hedrick J.L., Yang Y.Y. (2016). Co-delivery of drugs and plasmid DNA for cancer therapy. Adv. Drug Deliv. Rev..

[24] Navarro G., Pan J., Torchilin V.P. (2015). Micelle-like nanoparticles as carriers for DNA and siRNA. Mol. Pharm..

[25] Alinejad V., Hossein Somi M., Baradaran B., Akbarzadeh P., Atyabi F., Kazerooni H., SamadiKafil H., AghebatiMaleki L., Siah Mansouri H., Yousefi M. (2016). Co-delivery of IL17RB siRNA and doxorubicin bychitosan-based nanoparticles for enhanced anticancer efficacy in breast cancer cells. Biomed. Pharmacother..

[26] Wei W., Lv P.P., Chen X.M., Yue Z.G., Fu Q., Liu S.Y., Yue H., Ma G.H. (2013). Codelivery of mTERT siRNA and paclitaxel by chitosan-based nanoparticles promoted synergistic tumor suppression. Biomaterials.

[27] Li N., Huang C., Luan Y., Song A., Song Y., Garg S. (2016). Active targeting co-delivery system based onpH-sensitive methoxy-poly(ethylene glycol)2K-poly(epsilon-caprolactone)4K-poly(glutamic acid)1K forenhanced cancer therapy. J. Colloid Interface Sci..

[28] Pan J., Palmerston Mendes L., Yao M., Filipczak N., Garai S., Thakur G.A., Sarisozen C., Torchilin V.P. (2019). Polyamidoamine dendrimers-based nanomedicine for combination therapy with siRNAand chemotherapeutics to overcome multidrug resistance. Eur. J. Pharm. Biopharm..

[29] Wang X., Liow S.S., Wu Q., Li C., Owh C., Li Z., Loh X.J., Wu Y.L. (2017). Codelivery for Paclitaxel and Bcl-2 Conversion Gene by PHB-PDMAEMA Amphiphilic Cationic Copolymer for Effective Drug Resistant Cancer Therapy. Macromol. Biosci..

[30] Cheng Q., Du L., Meng L., Han S., Wei T., Wang X., Wu Y., Song X., Zhou J., Zheng S. (2016). The Promising Nanocarrier for Doxorubicin and siRNA Co-delivery by PDMAEMA-based Amphiphilic Nanomicelles. ACS Appl. Mater. Interfaces.

[31] Cheng H., Yang W., Chen H., Liu L., Gao F., Yang X., Jiang Q., Zhang Q., Wang Y. (2009). Surface modificationof mitoxantrone-loaded PLGA nanospheres with chitosan. Colloids Surf. B Biointerfaces.

[32] Wang L., Hao Y., Li H., Zhao Y., Meng D., Li D., Shi J., Zhang H., Zhang Z., Zhang Y. (2015). Co-delivery of doxorubicin and siRNA for glioma therapy by a brain targeting system: Angiopep-2-modifiedpoly(lactic-co-glycolic acid) nanoparticles. J. Drug Target.

[33] Cao N., Cheng D., Zou S., Ai H., Gao J., Shuai X. (2011). The synergistic effect of hierarchical assemblies of siRNA and chemotherapeutic drugs co-delivered into hepatic cancer cells. Biomaterials.

[34] Navarro G., Sawant R.R., Biswas S., Essex S., Tros de Ilarduya C., Torchilin V.P. (2012). P-glycoprotein silencingwith siRNA delivered by DOPE-modified PEI overcomes doxorubicin resistance in breast cancer cells. Nanomedicine.

[35] Huang H.Y., Kuo W.T., Chou M.J., Huang Y.Y. (2011). Co-delivery of anti-vascular endothelial growth factorsiRNA and doxorubicin by multifunctional polymeric micelle for tumor growth suppression. J. Biomed. Mater. Res. A.

[36] Knop K., Hoogenboom R., Fischer D., Schubert U.S. (2010). Poly(ethylene glycol) in drug delivery: Pros and cons as well as potential alternatives. Angew. Chem. Int. Ed..

[37] Zhou S., Deng X., Yang H. (2003). Biodegradable poly(epsilon-caprolactone)-poly(ethylene glycol) block copolymers: Characterization and their use as drug carriers for a controlled delivery system. Biomaterials.

[38] Zhang Z., Qu Q., Li J., Zhou S. (2013). The Effect of the Hydrophilic/Hydrophobic Ratio of Polymeric Micelles on their Endocytosis Pathways into Cells. Macromol. Biosci..

[39] Çırpanlı Y., Allard E., Passirani C., Bilensoy E., Lemaire L., Çalış S., Benoit J.P. (2011). Antitumoral activity ofcamptothecin-loaded nanoparticles in 9L rat glioma model. Int. J. Pharm..

[40] Hu J., Fu S., Peng Q., Han Y., Xie J., Zan N., Chen Y., Fan J. (2017). Paclitaxel-loaded polymeric nanoparticles combined with chronomodulated chemotherapy on lung cancer: In vitro and in vivo evaluation. Int. J.Pharm..

[41] Hong G., Yuan R., Liang B., Shen J., Yang X., Shuai X. (2008). Folate-functionalized polymeric micelle as hepatic carcinoma-targeted, MRI-ultrasensitive delivery system of antitumor drugs. Biomed. Microdevices.

[42] Wen X., Wu Q.P., Ke S., Ellis L., Charnsangavej C., Delpassand A.S., Wallace S., Li C. (2001). Conjugation with (111)In-DTPA-poly(ethylene glycol)improves imaging of anti-EGF receptor antibody C225. J. Nucl. Med..

[43] Lee H., Hoang B., Fonge H., Reilly R.M., Allen C. (2010). In vivo distribution of polymeric nanoparticles at the whole-body, tumor, and cellular levels. Pharm. Res..

[44] Guo J., Gao X., Su L., Xia H., Gu G., Pang Z., Jiang X., Yao L., Chen J., Chen H. (2011). Aptamer-functionalized PEG–PLGA nanoparticles for enhance danti-glioma drug delivery. Biomaterials.

[45] Shafiei-Irannejad V., Samadi N., Salehi R., Yousefi B., Rahimi M., Akbarzadeh A., Zarghami N. (2018). Reversion of Multidrug Resistance by Co-Encapsulation of Doxorubicin and Metformin in Poly(lactide-co-glycolide)-D-α-tocopheryl Polyethylene Glycol 1000 Succinate Nanoparticles. Pharm. Res..

[46] Wang H., Zhao Y., Wu Y., Hu Y.L., Nan K., Nie G., Chen H. (2011). Enhanced anti-tumor efficacy by co-deliveryof doxorubicin and paclitaxel with amphiphilic methoxy PEG-PLGA copolymer nanoparticles. Biomaterials.

[47] Xu X., Chen X., Wang Z., Jing X. (2009). Ultrafine PEG-PLA fibers loaded with both paclitaxel and doxorubicinhydrochloride and their in vitro cytotoxicity. Eur. J. Pharm. Biopharm..

[48] Duong H.H., Yung L.Y. (2013). Synergistic co-delivery of doxorubicin and paclitaxel using multi-functional micellesfor cancer treatment. Int. J. Pharm..

[49] Lv S., Tang Z., Li M., Lin J., Song W., Liu H., Huang Y., Zhang Y., Chen X. (2014). Co-delivery of doxorubicinand paclitaxel by PEG-polypeptide nanovehicle for the treatment of non-small cell lung cancer. Biomaterials.

[50] Matsumura Y. (2008). Polymeric Micellar Delivery Systems in Oncology. Jpn. J. Clin. Oncol..

[51] Wilson R.H., Plummer R., Adam J., Eatock M., Boddy A.V., Griffin M., Miller R., Matsumura Y., Shimizu T., Calvert H. (2008). Phase I and pharmacokinetic study of NC-6004, a new platinum entity of cisplatin-conjugated polymer forming micelles. Clin. Oncol..

[52] Plummer R., Wilson R.H., Calvert H., Boddy A.V., Griffin M., Sludden J., Tilby M.J., Eatock M., Pearson D.G., Ottley C.J. (2011). A Phase I clinical study of cisplatin-incorporated polymeric micelles (NC-6004) in patients with solid tumours T. Br. J. Cancer.

[53] Vega J., Ke S., Fan Z., Wallace S., Charsangavej C., Li C. (2003). Targeting doxorubicin to epidermal growth factor receptors bysite-specific conjugation of C225 to poly(L-glutamic acid)through a polyethylene glycol spacer. Pharm. Res..

[54] Yuan J.D., ZhuGe D.L., Tong M.Q., Lin M.T., Xu X.F., Tang X., Zhao Y.Z., Xu H.L. (2018). pH-sensitive polymericnanoparticles of mPEG-PLGA-PGlu with hybrid core for simultaneous encapsulation of curcumin anddoxorubicin to kill the heterogeneous tumour cells in breast cancer. Artif. Cells Nanomed. Biotechnol..

[55] Matsumura Y. (2008). Poly (amino acid) micelle nanocarriers in preclinical and clinical studies. Adv. Drug Deliv. Rev..

[56] Hamaguchi T., Matsumura Y., Suzuki M., Shimizu K., Goda R., Nakamura I., Nakatomi I., Yokoyama M., Kataoka K., Kakizoe T. (2005). NK105, a paclitaxel-incorporating micellar nanoparticle formulation, can extend *in vivo* antitumour activity and reduce the neurotoxicity of paclitaxel. Br. J. Cancer.

[57] Hamaguchi T., Kato K., Yasui H., Morizane C., Ikeda M., Ueno H., Muro K., Yamada Y., Okusaka T., Shirao K. (2007). A phase I and pharmacokinetic study of NK105, a paclitaxel-incorporating micellar nanoparticle formulation. Br. J. Cancer.

[58] Vilar G., Puche J.T., Albericio F. (2012). Polymers and drug delivery systems. Curr. Drug Deliv..

[59] Venne A., Li S., Mandeville R., Kabanov A., Alakhov V. (1996). Hypersensitizing effect of pluronic L61 on cytotoxic activity, transport and subcellular distribution of doxorubicin in multiple drug-resistant cells. Cancer Res..

[60] Valle J.W., Armstrong A., Newman C., Alakhov V., Pietrzynski G., Brewer J., Campbell S., Corrie P., Rowinsky E.K., Ranson M. (2010). A phase 2 study of SP1049C, doxorubicin in P-glycoprotein-targeting pluronics, in patients with advanced adenocarcinoma of the esophagus and gastroesophageal junction. Investig. New Drugs.

[61] Hu D., Chen L., Qu Y., Peng J., Chu B., Shi K., Hao Y., Zhong L., Wang M., Qian Z. (2018). Oxygen-generating Hybrid Polymeric Nanoparticles with Encapsulated Doxorubicin and Chlorin e6 for Trimodal Imaging-Guided Combined Chemo-Photodynamic Therapy. Theranostics.

[62] Jin M., Jin G., Kang L., Chen L., Gao Z., Huang W. (2018). Smart polymeric nanoparticles with pH-responsive and PEG-detachable properties for co-delivering paclitaxel and survivin siRNA to enhance antitumor outcomes. Int. J. Nanomed..

[63] Greenwald R.B., Pendri A., Conover C.D., Lee C., Choe Y.H., Gilbert C., Martinez A., Xia Y., Wu D., Hsue M. (1998). Camptothecin-20-PEG ester transport forms: The effect of spacer groups on antitumor activity. Bioorg. Med. Chem..

[64] Fraier D., Frigerio E., Brianceschi G., Casati M., Benecchi A., James C. (2000). Determination of MAG-Camptothecin, a new polymer-bound Camptothecin derivative, and free Camptothecin in dog plasma by HPLC with fluorimetricdetection. J. Pharm. Biomed. Anal..

[65] Singer J.W., Bhatt R., Tulinsky J., Buhler K.R., Heasley E., Klein P., James C. (2001). Water-soluble poly-(l-glutamic acid)–Gly-camptothecin conjugates enhance camptothecin stability and efficacy in vivo. J. Control. Release.

[66] Pastorino F., Loi M., Sapra P., Becherini P., Cilli M., Emionite L., Ribatti D., Greenberger L.M., Horak I.D., Ponzoni M. (2010). Tumor Regression and Curability of Preclinical Neuroblastoma Models by PEGylated SN38 (EZN-2208), a Novel Topoisomerase I Inhibitor. Clin. Cancer Res..

[67] Sapra P., Zhao H., Mehlig M., Malaby J., Kraft P., Longley C., Greenberger L.M., Horak I.D. (2008). Novel Delivery of SN38 Markedly Inhibits Tumor Growth in Xenografts, Including a Camptothecin-11–Refractory Model. Clin. Cancer Res..

[68] Crozier J.A., Advani P.P., Plant B.L., Anthony T.H., Jaslowski J., Moreno-Aspitia A., Perez E.A. (2016). N0436 (Alliance): A phase II trial of irinotecan plus cetuximab in patients with metastatic breast cancer previously exposed to anthracycline and/or taxane-containing therapy. Clin. Breast Cancer.

[69] Duncan R., Vicent M.J. (2010). Do HPMA copolymer conjugates have a future as clinically useful nanomedicines? A critical overview of current status and future opportunities. Adv. Drug Deliv. Rev..

[70] Seymour L.W., Ferry D.R., Kerr D.J., Rea D., Whitlock M., Poyner R., Boivin C., Hesslewood S., Twelves C., Blackie R. (2009). Phase II studies of polymer-doxorubicin (PK1, FCE28068) in the treatment of breast, lung and colorectal cancer. Int. J. Oncol..

[71] Terwogt J.M.M., ten BokkelHuinink W.W., Schellens J.H.M., Schot M., Mandjes I., Zurlo M., Rocchetti M., Rosing H., Koopman F.M., Beijnen J.H. (2001). Phase I clinical and pharmacokinetic study of PNU166945, a novel water-soluble polymer-conjugated prodrug of paclitaxel. Anticancer Drugs.

[72] Campone M., Rademaker-Lakhai J.M., Bennouna J., Howell S.B., Nowotnik D.P., Beijnen J.H., Schellens J.H. (2007). Phase I and pharmacokinetic trial of AP5346, a DACH-platinum-polymer conjugate, administered weekly for three out of every 4 weeks to advanced solid tumor patients. Cancer Chemother. Pharmacol..

[73] Rice J.R., Howell S.B. (2004). AP-5346. Drugs Future.

[74] Kelland L. (2007). Broadening the clinical use of platinum drug–based chemotherapy with new analogues. ExpertOpin. Investig. Drugs.

[75] Nowotnik D.P., Cvitkovic E. (2009). ProLindac™(AP5346): A review of the development of an HPMA DACH platinum Polymer Therapeutic. Adv. Drug Deliv. Rev..

[76] Pan J., Rostamizadeh K., Filipczak N., Torchilin V. (2019). Polymeric Co-Delivery Systems in Cancer Treatment: An Overview on Component Drugs Dosage Ratio Effect. Molecules.

[77] Das L., Vinayak M. (2014). Long-term effect of curcumin down-regulates expression of tumor necrosis factor-alphaand interleukin-6 via modulation of E26 transformation-specific protein and nuclear factor-κB transcription factors in livers of lymphoma bearing mice. Leuk. Lymphoma.

[78] Tuorkey M.J. (2014). Curcumin a potent cancer preventive agent: Mechanisms of cancer cell killing. Interv. Med.Appl. Sci..

[79] Wang B.L., Shen Y.M., Zhang Q.W., Li Y.L., Luo M., Liu Z., Li Y., Qian Z.Y., Gao X., Shi H.S. (2013). Codelivery of curcumin and doxorubicin by MPEG-PCL results in improved efficacy of systemically administered chemotherapy in mice with lung cancer. Int. J. Nanomed..

[80] Duan J., Mansour H.M., Zhang Y., Deng X., Chen Y., Wang J., Pan Y., Zhao J. (2012). Reversion of multidrug resistance by co-encapsulation of doxorubicin and curcumin in chitosan/poly(butyl cyanoacrylate)nanoparticles. Int. J. Pharm..

[81] Guo O., Li X., Yang Y., Wei J., Zhao Q., Luo F., Qian Z. (2014). Enhanced 4T1 breast carcinoma anticancer activity by co-delivery of doxorubicin and curcumin with core-shell drug-carrier based on heparin modified poly(L-lactide) grafted polyethylenimine cationic nanoparticles. J. Biomed. Nanotechnol..

[82] Danson S., Ferry D., Alakhov V., Margison J., Kerr D., Jowle D., Brampton M., Halbert G., Ranson M. (2004). Phase I dose escalation and pharmacokinetic study of pluronicpolymer-bound doxorubicin (SP1049C) in patients with ad-vanced cancer. Br. J. Cancer.

[83] Wang Y., Yu L., Han L., Sha X., Fang X. (2007). Difunctional Pluroniccopolymer micelles for paclitaxel delivery: Synergistic effect of folate-mediated targeting and Pluronic-mediated overcoming multidrugresistance in tumor cell lines. Int. J. Pharm..

[84] Chen Y., Zhang W., Huang Y., Gao F., Sha X., Fang X. (2015). Pluronic-based functional polymeric mixed micellesfor co-delivery of doxorubicin and paclitaxel to multidrug resistant tumor. Int. J. Pharm..

[85] Ma Y., Fan X., Li L. (2016). pH-sensitive polymeric micelles formed by doxorubicin conjugated prodrugs forco-delivery of doxorubicin and paclitaxel. Carbohydr. Polym..

[86] Wang H., Agarwal P., Zhao S., Xu R.X., Yu J., Lu X., He X. (2015). Hyaluronic acid-decorated dual responsivenanoparticles of Pluronic F127, PLGA, and chitosan for targeted co-delivery of doxorubicin and irinotecan toeliminate cancer stem-like cells. Biomaterials.

[87] Tang S., Yin Q., Su J., Sun H., Meng Q., Chen Y., Chen L., Huang Y., Gu W., Xu M. (2015). Inhibition ofmetastasis and growth of breast cancer by pH-sensitive poly (beta-amino ester) nanoparticles co-deliveringtwo siRNA and paclitaxel. Biomaterials.

[88] Pasut G., Veronese F.M. (2009). PEG conjugates in clinical development or use as anticancer agents: An overview. Adv. Drug Deliv. Rev..

[89] Kopecek J., Kopeckova P. (2010). HPMA copolymers: Origins, early developments, present, and future. Adv. Drug Deliv. Rev..

[90] Hopewel W., Duncan R., Wilding D., Chakrabarti K. (2001). Preclinical evaluation of the cardiotoxicity of PK2: A novel HPMA copolymer–doxorubicin–galactosamine conjugate antitumouragent. Hum. Exp. Toxicol..

[91] Julyan P.J., Seymour L.W., Ferry D.R., Daryani S., Boivin C.M., Doran J., David M., Anderson D., Christodoulou C., Young A.M. (1999). Preliminary clinical study of the distribution of HPMA copolymers bearing doxorubicin and galactosamine. J. Control. Release.

[92] Seymour L.W., Ferry D.R., Anderson D., Hesslewood S., Julyan P.J., Poyner R., Doran J., Young A.M., Burtles S., Kerr D.J. (2002). Hepatic Drug Targeting: Phase I Evaluation of Polymer-Bound Doxorubicin. J. Clin. Oncol..

[93] Bae Y., Nishiyama N., Fukushima S., Koyama H., Yasuhiro M., Kataoka K. (2005). Preparation and biological characterization of polymeric micelle drug carriers with intracellular pH-triggered drug release property: Tumor permeability, controlled subcellular drug distribution, and enhanced *invivo* antitumor efficacy. Bioconjug.Chem..

[94] Xiong X.B., Ma Z., Lai R., Lavasanifar A. (2010). The therapeutic response to multifunctional polymeric nano-conjugates in the targeted cellular and subcellular delivery of doxorubicin. Biomaterials.

[95] Wu P., Opadele A.E., Onodera Y., Nam J. (2019). Targeting Integrins in Cancer Nanomedicine: Applications in Cancer Diagnosis and Therapy. Cancers.

[96] Shi C., Guo X., Qu Q., Tang Z., Wang Y., Zhou S. (2014). Actively targeted delivery of anticancer drug to tumor cells by redox-responsive star-shaped micelles. Biomaterials.

[97] Chen W., Shah L.A., Yuan L., Siddiq M., Hu J., Yang D. (2015). Polymer–paclitaxel conjugates based on disulfide linkers for controlled drug release. RSC Adv..

